# Are Branded and Generic Extended-Release Ropinirole Formulations Equally Efficacious? A Rater-Blinded, Switch-Over, Multicenter Study

**DOI:** 10.1155/2014/158353

**Published:** 2014-08-26

**Authors:** Edit Bosnyák, Mihály Herceg, Endre Pál, Zsuzsanna Aschermann, József Janszky, Ildikó Késmárki, Sámuel Komoly, Kázmér Karádi, Tamás Dóczi, Ferenc Nagy, Norbert Kovács

**Affiliations:** ^1^Department of Neurology, University of Pécs, Rét Utca 2, Pécs 7623, Hungary; ^2^Department of Neurology, Kaposi Mór County Hospital, Tallián Gyula Utca 20-32, Kaposvár 7400, Hungary; ^3^MTA-PTE Clinical Neuroscience MR Research Group, Rét Utca 2, Pécs 7623, Hungary; ^4^Department of Neurology, Health Center of City of Pécs, Dr. Veress Endre Utca 2, Pécs 7633, Hungary; ^5^Institute of Behavioral Sciences, University of Pécs, Szigeti Utca 12, Pécs 7624, Hungary; ^6^Department of Neurosurgery, University of Pécs, Rét Utca 2, Pécs 7623, Hungary

## Abstract

The aim of this study was to compare the efficacy of the branded and a generic extended-release ropinirole formulation in the treatment of advanced Parkinson's disease (PD). Of 22 enrolled patients 21 completed the study. A rater blinded to treatment evaluated Unified Parkinson's Disease Rating Scale, Fahn-Tolosa-Marin Tremor Rating Scale, Nonmotor Symptoms Assessment Scale, and a structured questionnaire on ropinirole side effects. Besides, the patients self-administered EQ-5D, Parkinson's Disease Sleep Scale (PDSS-2), and Beck Depression Inventories. Branded and generic ropinirole treatment achieved similar scores on all tests measuring severity of motor symptoms (primary endpoint, UPDRS-III: 27.0 versus 28.0 points, *P* = 0.505). Based on patient diaries, the lengths of “good time periods” were comparable (10.5 and 10.0 hours for branded and generic ropinirole, resp., *P* = 0.670). However, generic ropinirole therapy achieved almost 3.0 hours shorter on time without dyskinesia (6.5 versus. 9.5 hours, *P* < 0.05) and 2.5 hours longer on time with slight dyskinesia (3.5 versus. 1.0 hours, *P* < 0.05) than the branded ropinirole did. Except for gastrointestinal problems, nonmotor symptoms were similarly controlled. Patients did not prefer either formulation. Although this study has to be interpreted with limitations, it demonstrated that both generic and branded ropinirole administration can achieve similar control on most symptoms of PD.

## 1. Introduction

Parkinson's disease (PD) is a neurodegenerative disease having major burdens on both the families and the healthcare system. Although most symptoms of PD have an excellent response to dopamine replacement therapies (e.g., levodopa and dopamine agonists, DA) in the early phase, the response to medication may become progressively inconsistent as the disease progresses. As the therapeutic window becomes narrower, long-term complication of dopaminergic treatment develops. The pharmacological management of wearing off, fluctuations, and dyskineasias (both chorea and dystonia) may be challenging. Therefore, patients with advanced PD usually require complex drug combinations, more frequent dosing, and usually increased dosages [1, 2].

Although dopaminergic medications are not considered as very expensive compared to other pharmacological agents, the long treatment duration and the frequent need to use complex combinations [1] makes the overall cost on these drugs high. The economic crisis and aging society encourage social security providers to reduce healthcare-related expenses, for example, by the application of generic drugs as substitutes for branded agents [3].

Medicinal products are considered to be pharmaceutically equivalent if they contain the same amount of the same active substance(s) in the same dosage forms that meet the same or comparable standards. Pharmaceutical equivalence does not necessarily imply bioequivalence as differences in the excipients and/or the manufacturing process can lead to faster or slower dissolution and/or absorption.

On the basis of European Medicines Agency (EMA) recommendations, two medicinal products containing the same active substance are considered bioequivalent if they are pharmaceutically equivalent or pharmaceutical alternatives (medicinal products with same active substance but in different salts or esters, etc.) and their bioavailabilities (rate and extent) after administration in the same molar dose lie within acceptable predefined limits [4]. The current concept is that pharmaceutical equivalence and bioequivalence are used as a surrogate measure for therapeutic effect [5]. Similar guidelines exist worldwide. For example, the Food and Drug Administration (FDA) requires almost the same parameters for licensing a generic drug [6].

Based on the assumption that bioequivalence equals therapeutic equivalence, generic and branded drugs can be used interchangeably. The regulatory limits applied in bioequivalence studies require that the areas under the drug concentration (AUC) versus time curves must be within 90% confidence intervals and the maximum plasma concentrations (*C*_max⁡_) must fall within 80–125% [4]. This rule implies that the difference theoretically could be as much as a 56% increase or 36% decrease in bioavailability when switching a patient from one generic to another generic formulation [4, 7].

It is well known that blood levels of levodopa (and DA) correlate with the appearance of both motor and nonmotor symptoms PD [8] and consequently can impact quality of life [9]. In particular in the advanced stage of PD, neither the too low nor the too high plasma concentration of dopaminergic medication is appropriate. The too low plasma levels could induce or worsen OFF periods, whereas the high levels could elicit or worsen dyskinesia [10]. Therefore, one could assume that the demonstration of an 80–125% bioequivalence for a generic drug compared to the branded medication might be too wide and could interfere with the PD symptoms.

Based on this assumption and the previously described correlation between the late dopaminergic side-effects and plasma concentrations, one can argue that dopaminergic drugs licensed for advanced PD should be considered as narrow therapeutic index drugs (NTIDs), in which group the acceptance interval for AUC is tightened by the current regulations to 90.00–111.11% in bioequivalence studies [11]. Although the current EMA regulations imply specific rules for NTIDs [12], these regulations are not applied for dopaminergic drugs. According to the revised guideline on the investigation of bioequivalence of EMA published in 2010, “It is not possible to define a set of criteria to categorize drugs as NTIDs and it must be decided case by case if an active substance is an NTID based on clinical considerations [11].”

Further possible issues in the generic drug prescription for advanced PD might be raised. One possible concern might be that currently bioequivalence measurements for dopaminergic medications are not performed on PD patients. Whereas the development of a branded medication demands the establishment of pharmacokinetics, efficacy, safety, and tolerability on both healthy and target patient population, the licensing of a new generic drug requires only the demonstration of bioequivalence with the branded counterpart, which is done only in healthy subjects [13]. Therefore, the gastrointestinal features of PD might also have an—uninvestigated—impact on the efficacy of generic drugs. Whereas neither the current nor the proposed [14] guideline of EMA on orally modified-release agents demands so, the product-specific guidance on ropinirole-hydrochloride of the Food and Drug Administration requires bioequivalence studies in both fasting and fed conditions.

There are very few available reports from either patients or physicians that address the question of generic drug-usage in PD from clinical perspectives [13]. The majority of these data compared the original levodopa formulations to generic ones and demonstrated that the minority of patients (31%) did not tolerate generic formulations [13, 15].

Recently, a study compared the pharmaceutical quality of seven generic levodopa/benserazide hydrochloride combination products marketed in Germany with the original product (Madopar) [16]. All generic products (100 mg/25 mg formulations) were tested against Madopar 125 tablets. They assessed the disintegration and dissolution, content, identity, and amounts of impurities along with standard physical and chemical laboratory. The authors state that each of the seven generic products had one or two parameters outside the specifications. Deviations for the active ingredients ranged from +8.4% (benserazide) to −7.6% (levodopa), whereas degradation products were measured in apparent excess (+26.5%) in one capsule formulation [16]. However, disintegration time and dissolution for levodopa and benserazide hydrochloride at 30 min were within specifications with some outliers [16]. The authors concluded that deviations for the active ingredients may go unnoticed by a new user of the generic product but may entail clinical consequences when switching from original to generic during a long-term therapy. Based on their opinion, degradation products may also pose a safety concern [16].

Even less data is available on the generic formulations of DAs. Because modified-release (usually extended-release) formulations of various DAs are available on the market, another issue might be raised concerning the length of clinical action. Whereas branded extended-release dopamine-agonists are claimed to have beneficial effects lasting for 24 hours, EMA and other regulators do not require any clinical evaluation of duration of action before licensing new extended-release generic drug formulations. Instead of clinical studies, the performance of much cheaper dissolution tests is required [14].

The aim of the present study was to compare the clinical efficacy of the branded extended-release ropinirole (Requip modutab, GlaxoSmithKline) with an also extended-release generic comparator (Ralnea, Krka, Slovenia). We assumed that any significant differences between these two formulations could produce altered severity of either motor or nonmotor symptoms of PD.

## 2. Methods

### 2.1. Patients

Twenty-two Parkinson's disease (PD) patients with fluctuation were enrolled to our study (age: 69.3 ± 10.9 years, sex: 17 males, disease duration: 6.5 ± 2.9 years, and type of PD: 17 rigid-akinetic, 5 mixed). All of them had advanced PD with long-term dopaminergic side-effects (wearing off and fluctuation) and received levodopa (423.8 ± 249.3 mg) and ropinirole (15.0 ± 5.6 mg) combination therapy. Each patient took extended-release (retard) formulation of ropinirole once-a-day, in the morning.

Inclusion criteria for participation were the following:

- fulfillment of the UK Brain Bank clinical diagnostic criteria for PD [17],
- receiving stable (≥8 mg/day) dosage of extended-release ropinirole >3 months,
- stable symptoms of PD before enrollment,
- signed written consent according to the approval of Regional Ethical Board.

Exclusion criteria were the following:

- presence of other disorders capable of producing tremor (e.g., hyperthyroidism),
- suspicion of any Parkinson Plus syndromes or secondary parkinsonism,
- abnormal brain MRI (e.g., hydrocephalus, brainstem atrophy, and metal deposition),
- implanted deep brain stimulator,
- levodopa/carbidopa intestinal gel infusion therapy,
- history of drug abuse,
- presence of any contraindications for ropinirole administration,
- presence or history of impulsive control disorder,
- suspicion of psychogenic symptoms,
- presence of any disorders capable of interfering with the study (e.g., severe heart failure, severe arthritis, severe liver problems, or dementia).

Patients were examined at Department of Neurology, University of Pécs (Pécs, Hungary) and Kaposi Mór County Hospital (Kaposvár, Hungary). The study protocol was approved by the Regional Ethical Board (4230.316-2470/KK15/2011). Our examination was an investigator initiated and noncommercial study. Requip modutab and Ralnea medication were obtained from GSK and Krka pharmaceutical companies, respectively. None of the manufacturers had any influence on the present study.

### 2.2. Methods

Our a priori hypothesis was that the generic ropinirole has noninferiority compared to the branded one. The study had a rater-blinded and crossover design lasting for 3 months. At enrollment and 1 month later (Visits 1 and 2), the patients received stable dosage of branded ropinirole (Requip modutab). After completing the tests of Visit 2, the patients switched to same dosage of generic ropinirole; therefore, we evaluated the clinical effects of Ralnea at Visits 3 and 4. Except for switching from Requip to Ralnea, the patients received unchanged medication and dosing throughout the study.

Before enrollment, neuropsychological tests (Mini-Mental Status Examination, Mattis Dementia Rating Scale) were applied to exclude demented patients [18]. We used the Hungarian validated cut-off score of 125 to exclude patients [18].

At each visit, the motor symptoms (Unified Parkinson's Disease Rating Scale part 3, UPDRS-3; Fahn-Tolosa-Marin Tremor Rating Scale part A, FTMTRS-A, and modified Hoehn-Yahr Stage, HYS) were tested at the same part of the day. These tests were scheduled 1.5 hours after the intake of last levodopa dose. We videotaped the application of these motor tests according to our video protocol [19]. After the accomplishment of the study, a rater specialized in movement disorders and blinded to the treatment (EB) reevaluated these digitalized and anonymous video recordings. The scores of these motor tests (except for rigidity subscore of UPDRS-3) were obtained during this blinded reevaluation [19]. We considered the severity of motor symptoms measured by UPDRS-3 as the primary endpoint of the study, whereas FTMTRA-A and HYS served as secondary endpoints.

A trained Parkinson nurse obtained the rest of UPDRS (parts 1, 2, and 4), FTMTRS (parts B and C), Nonmotor Symptom Assessment Scale (NMSS), activity of daily living (Schwab and England, ADL), a structured questionnaire on ropinirole side-effects, and Montgomery Asberg Depression Rating Scale (MADRS). Besides, the patients also self-administered the EQ-5D quality of life instrument [20], Parkinson's Disease Sleep Scale (PDSS-2), Epworth Sleepiness Scale (ESS), and Beck Depression Inventory (BDI).

We asked our patients to keep patient diaries at least 2 days before each visit to assess ON-time without dyskinesia, ON-time with slight dyskinesia, ON-time with severe dyskinesia, OFF-time, and daytime sleeping time [19]. “Good time” was defined as the sum of ON periods without dyskinesia and slight, nondisturbing dyskinesia [21–23]. During the last (fourth) visit we also obtained a structured inventory on the comparison of branded and generic ropinirole.

### 2.3. Statistical Methods

Because most of the variables did not follow the normal distribution, nonparametric tests were applied. Kruskal-Wallis test was assessed to evaluate any differences between Visits 1–4 except for HYS where Chi-square test was calculated by using SPSS Software (IBM Inc, version 19, IL). Wilcoxon's signed rank test was used during the comparison of two visits for continuous variables, whereas McNemar test for dichotomous variables and Chi-square for ordinal variables. The level of statistical significance was set at 0.05.

## 3. Results

Twenty-one patients completed the study; only their data were used for further analyses. One patient withdrew from the study because he moved to another city and consequently became unable to carry on with the protocol.

The result of Mini-Mental Status Examination was 26.6 ± 2.6 points, whereas the average score on Mattis Dementia Rating Scale [18] was 133.0 ± 7.8 points.

Before further analyses, we compared the results of Visit 1 with Visit 2. Because none of the examined variables changed significantly between Visits 1-2, we assumed that our patients did not experience any changes (either worsening or improvement) during the Requip modutab phase. Similarly we did not identify any changes during the stable Ralnea phase (between Visits 3-4).

### 3.1. Motor Symptoms

As far as the motor symptoms were concerned, we did not observe any differences between each visit (UPDRS-3, FTMTRS-A, HYS, *P* > 0.05, Table 1).

### 3.2. Patient Diary

The analysis of the patient diary revealed that the length of ON periods without dyskinesia decreased (worsened) from 9.5 hours to 6.5 hours after switching from branded to generic ropinirole (*P* = 0.01, Table 1). Simultaneously, the length of ON periods with slight dyskinesia increased from 1.0 to 3.5 hours (*P* < 0.05). Consequently, the length of “good time periods” remained unchanged between the branded and generic ropinirole treatment phase (10.5 versus 10.0 hours, *P* > 0.05, Table 1). Similarly, the lengths of OFF periods, ON periods with severe dyskinesia, daytime sleep, and nighttime sleep periods did not change significantly (Table 1).

### 3.3. Other Tests

Among the nonmotor tests (PDSS-2, ESS, NMSS, EQ-5D, ADL, MADRS, and BDI) only the gastrointestinal section of NMSS showed worsening during generic ropinirole treatment (from 4.0 to 8.0 points, *P* < 0.05, Table 1). However, the side-effect profile of both medications was comparable on the structured ropinirole side-effects questionnaire (Table 2). The analysis of the after completion feedback forms revealed that similar number of patients requested Requip and Ralnea medication after completion of the study (12 versus 9, resp., *P* = 0.513, Table 3). Six months later 8 patients were on branded, whereas 13 subjects were on generic formulation (*P* = 0.275).

## 4. Discussion

To our knowledge, our study is the first direct comparison of the branded and a generic extended-release ropinirole in the treatment of advanced PD. Although, our results demonstrated that both generic ropinirole and branded ropinirole usage achieved similar motor-symptom control measured by UPDRS-3 and met the criteria for the primary endpoint, some differences could also be revealed. The usage of generic formulation was associated with higher rate of gastrointestinal symptoms measured by NMSS, which was inconsistent with the results of the ropinirole side-effects questionnaire. Besides, the length of ON periods without dyskinesia was 3.0 hours shorter and the length of ON periods with slight and nondisturbing dyskinesia was almost 2.5 hours longer during the generic medication usage. Despite the above mentioned differences in gastrointestinal symptoms and patient diary periods, the patients did not prefer either formulation after completing the study protocol. This might be due the fact that the time with “good periods” [21–23] remained comparable between the Requip and Ralnea treatments (10.5 versus 10.0 hours, resp.) and the severity of motor and most nonmotor symptoms was also similar.

The clinical significance of the increased ON time with slight dyskinesia during the administration of generic ropinirole remains unknown. We might assume that this phenomenon is probably due to slightly increased level of serum ropinirole during the periods of dyskinesia. Because the results of quality of life scales did not differ between the branded and generic ropinirole administration, its clinical relevance on the patients' everyday life might be minimal.

One of our key findings was that the generic formulation (Ralnea) achieved similar control on the motor and nonmotor symptoms of PD and both formulations had similar safety and side-effect profile with the exception of gastrointestinal problems. Although the NMSS score on gastrointestinal problems significantly increased during the generic ropinirole treatment phase, the patient reported side-effect profile was similar in both generic and branded ropinirole treatment phases.

This basically positive outcome may also be verified by the clinical practice of the authors, because we have not observed any serious clinical issues with this generic ropinirole formulation since their introduction to the Hungarian market in 2011. Despite similar number of patients choosing the branded and the generic formulations of ropinirole therapy immediately after completion of the study, 6 months later somewhat more patients were on generic formulation medication. In the background of this tendency, the increased financial burden of patients may be suspected. As the generic ropinirole formulations spread into the Hungarian market, the reimbursement of the branded formulation was lowered by the government-based healthcare system. Therefore the end-user price of branded ropinirole became increased compared to its comparators. In our opinion, the increased patients' costs might have been the main reason why increasingly more patients chose the generic ropinirole treatment.

Although studies of generic versus branded products are very difficult to perform across a large population of patients, the authors are aware of the major limitations of the study: because our study was not a randomized and double-blind examination, the patients' preference and beliefs might have biased the results. Theoretically we could have applied overcapsulation to ensure double-blind evaluation, but we were unable to organize such study without appropriate funding. Therefore, we applied a blinded-rater approach and planned four visits (2 with branded and 2 with generic drug usage) to minimize possible biases.

Another limitation of the study is the lack of pharmacokinetic data. Because our study was a nonsponsored and investigator-initiated examination, we did not have any opportunity to study the pharmacokinetics of the branded and generic ropinirole.

## Figures and Tables

**Table 1:** Results of tests measuring various motor and nonmotor symptoms.

		Requip (Visit 2)			Ralnea (Visit 4)			Significance
		Median	25th percentile	75th percentile	Median	25th percentile	75th percentile	
UPDRS and HYS	UPDRS part I	2.0	2.0	4.0	3.0	2.5	5.0	0.566
	UPDRS part II	15.0	12.0	17.0	15.0	9.5	19.0	0.928
	UPDRS part III	27.0	20.5	31.0	28.0	23.0	33.5	0.505
	UPDRS part IV	8.0	5.0	10.0	7.0	5.0	9.5	0.857
	UPDRS total score	52.0	40.5	59.5	55.0	42.5	66.5	0.776
	HYS	2.0	2.0	2.5	2.0	2.0	2.8	0.785

Tremor	FTMTRS part A	5.0	3.5	9.0	5.0	4.0	7.0	0.919
	FTMTRS part B	10.0	9.0	10.5	12.0	9.5	13.0	0.502
	FTMTRS part C	10.0	8.0	10.5	9.0	6.0	11.0	0.810
	FTMTRS total score	25.0	21.0	29.0	25.0	22.0	28.5	0.918

Patient diary	**ON time without dyskinesia (hours)**	**9.5**	**7.5**	**12.0**	**6.5**	**3.5**	**8.4**	**0.010**
	**ON time with slight dyskinesia (hours)**	**1.0**	**0.0**	**2.5**	**3.5**	**0.5**	**4.5**	**0.041**
	“Good” time	10.5	7.5	13.5	10.0	8.0	13.0	0.670
	ON time with severe dyskinesia (hours)	0.5	0.0	1.0	0.5	0.0	1.5	0.612
	OFF time (hours)	4.5	3.5	7.5	4.5	1.5	11.5	0.778
	Daytime sleeping hours	0.5	0.0	1.5	0.5	0.0	1.0	0.828
	Nighttime sleeping hours	7.5	6.8	8.3	7.0	6.3	7.8	0.468

Nonmotor symptoms	NMS section I (cardiovascular subscore)	2.0	0.0	6.0	3.0	0.0	7.0	0.633
	NMS section II (sleep subscore)	17.0	11.0	26.0	20.0	15.0	26.0	0.993
	NMS section III (mood subscore)	11.0	3.5	20.0	12.0	4.5	25.0	0.907
	NMS section IV (hallucinations subscore)	0.0	0.0	8.0	0.0	0.0	4.0	0.974
	NMS section V (memory subscore)	10.0	3.0	18.5	12.0	6.0	21.0	0.523
	**NMS section VI (gastrointestinal subscore)**	**4.0**	**2.0**	**7.5**	**8.0**	**4.0**	**11.0**	**0.016**
	NMS section VII (urinary system subscore)	13.0	9.0	19.5	14.0	9.0	20.5	0.878
	NMS section VIII (sexual subscore)	2.0	0.0	10.0	2.0	0.0	5.0	0.197
	NMS section IX (miscellaneous subscore)	1.0	0.0	9.0	1.0	0.0	7.0	0.251
	NMS total score	85.0	47.0	103.5	90.0	59.5	115.5	0.939

Sleep and sleepiness	PDSS-2 motor subscore	3.0	0.0	7.0	2.0	1.0	7.0	0.397
	PDSS-2 Parkinsonian symptoms subscore	4.0	2.0	5.5	3.0	1.0	5.5	0.657
	PDSS-2 disturbed sleep subscore	6.0	3.5	9.5	7.0	3.5	11.0	0.334
	PDSS-2 total score	12.0	7.5	18.5	10.0	8.0	23.0	0.224
	ESS Total	7.0	6.0	12.0	7.0	5.5	12.0	0.567

Depression	BDI total score	10.0	5.5	15.5	9.0	5.0	14.0	0.858
	MADRS total score	10.0	5.5	15.0	11.0	7.5	15.5	0.623

QoL	EQ-5D	0.8	0.7	0.9	0.8	0.7	0.9	0.895
	EQ-VAS	70.0	54.0	80.0	63.0	50.0	76.0	0.625
	ADL (Schwab-England)	80.0	75.0	90.0	80.0	70.0	90.0	0.965
	Clinical global impression-severity	3.0	2.0	4.0	3.0	1.5	3.5	0.647
	Clinical global impression-improvement	4.0	4.0	4.0	4.0	4.0	5.0	0.199

ADL = activities of daily living (Schwab and England Scale). BDI = Beck Depression Inventory; ESS = Epworth Sleepiness Scale; EQ-5D = the EuroQol instrument for detecting health outcome; EQ-VAS = visual analogue scale included in EQ-5D; HYS = Hoehn-Yahr Stage; FTMTRS = Fahn-Tolosa-Marin Tremor Rating Scale; MADRS = Montgomery Asberg Depression Rating Scale; NMSS = Nonmotor Symptom Assessment Scale; PDSS-2 = Parkinson's Disease Sleep Scale 2nd version; UPDRS = Unified Parkinson's Disease Rating Scale. For calculating significance Kruskal-Wallis test was applied except for HYS and Clinical Global Impression scales where Chi-square test was used (because of being an ordinal variable).

**Table 2:** Side-effect profile.

	Requip (present/absent)	Ralnea (present/absent)	McNemar test (*P* value)
Orthostatic hypotension	9/12	9/12	1.000
Daytime sleepiness	18/3	19/2	1.000
Dizziness	8/13	8/13	1.000
Confusion	0/21	2/19	0.500
Dyskinesia	14/7	17/4	0.219
Hallucinations or pseudohallucinations	4/17	3/18	1.000
Sexual disturbances	1/20	2/19	1.000
Abdominal pain/discomfort	2/19	7/14	0.125
Nausea	1/20	3/18	0.500
Constipation	7/14	6/15	1.000
Oedema	13/8	10/11	0.250
Impulse control disorder	0/21	0/21	1.000

**Table 3:** Results of the poststudy patient questionnaire.

Question	Responses (Requip/Ralnea/Similar)	Chi-square test
Overall which formulation had better efficacy?	9/4/8	0.368
Which formulation had better control on motor symptoms?	10/3/8	0.156
Which formulation had better control on nighttime symptoms?	5/6/10	0.368
Which formulation had better control on nonmotor symptoms?	8/0/13	0.275
Which formulation would you like to receive after the completion of the study?	12/9	0.513

## Abbreviations

ADL: Activities of daily living (Schwab and England Scale)
AUC: Area under the drug concentration curve
BDI: Beck Depression Inventory
DA: Dopamine agonists
EMA: European Medicines Agency
ESS: Epworth Sleepiness Scale
EQ-5D: The EuroQol instrument for detecting health outcome
EQ-VAS: visual analogue scale included in EQ-5D
HYS: Hoehn-Yahr Stage
FTMTRS: Fahn-Tolosa-Marin Tremor Rating Scale
MADRS: Montgomery Asberg Depression Rating Scale
NMSS: Nonmotor Symptom Assessment Scale
NTID: Narrow therapeutic index drugs
PD: Parkinson's disease
PDSS-2: Parkinson's Disease Sleep Scale 2nd version
UPDRS: Unified Parkinson's Disease Rating Scale.
